# Pediatric Intensive Care Hybrid-Style Clinical Round During COVID-19 Pandemic: A Pilot Study

**DOI:** 10.3389/fped.2021.720203

**Published:** 2021-08-19

**Authors:** Mohamad-Hani Temsah, Ali Alhboob, Noura Abouammoh, Ayman Al-Eyadhy, Fadi Aljamaan, Fahad Alsohime, Majed Alabdulhafid, Ahmad Ashry, Ahmad Bukhari, Omer ElTahir, Amr Jamal, Rabih Halwani, Khalid Alhasan, Adi Alherbish, Reem Temsah, Jaffar A. Al-Tawfiq, Mazin Barry

**Affiliations:** ^1^College of Medicine, King Saud University, Riyadh, Saudi Arabia; ^2^Pediatric Intensive Care Unit, Department of Pediatrics, King Saud University Medical City, King Saud University, Riyadh, Saudi Arabia; ^3^Department of Family and Community Medicine, King Saud University, Riyadh, Saudi Arabia; ^4^Critical Care Department, King Saud University Medical City, Riyadh, Saudi Arabia; ^5^Department of Medicine and Clinical Pharmacology, University of Medical Sciences and Technology (UMST), Khartoum, Sudan; ^6^Evidence-Based Health Care and Knowledge Translation Research Chair, King Saud University, Riyadh, Saudi Arabia; ^7^Sharjah Institute of Medical Research, University of Sharjah, Sharjah, United Arab Emirates; ^8^Department of Clinical Sciences, College of Medicine, University of Sharjah, Sharjah, United Arab Emirates; ^9^College of Pharmacy, Alfaisal University, Riyadh, Saudi Arabia; ^10^Specialty Internal Medicine and Quality Department, Johns Hopkins Aramco Healthcare, Dhahran, Saudi Arabia; ^11^Infectious Disease Division, Department of Medicine, Indiana University School of Medicine, Indianapolis, IN, United States; ^12^Infectious Disease Division, Department of Medicine, Johns Hopkins University School of Medicine, Baltimore, MD, United States; ^13^Division of Infectious Diseases, Department of Internal Medicine, College of Medicine, King Saud University and King Saud University Medical City, Riyadh, Saudi Arabia

**Keywords:** PICU hybrid rounds, PICU videoconferencing through Zoom, PICU Zoom teleconferencing, PICU multidisciplinary virtual round, Zoom clinical rounds

## Abstract

**Objectives:** With the evolving COVID-19 pandemic and the emphasis on social distancing to decrease the spread of SARS-CoV-2 among healthcare workers (HCWs), our pediatric intensive care unit (PICU) piloted the integration of Zoom meetings into clinical rounds. We aimed to explore the feasibility of these hybrid virtual and physical clinical rounds for PICU patients.

**Design:** Mixed quantitative and qualitative deductive thematic content analysis of narrative responses.

**Setting:** PICU, single tertiary-care academic center.

**Participants:** Multidisciplinary PICU HCWs.

**Interventions:** Integration of Zoom meeting into clinical daily PICU rounds.

**Measurements:** For the quantitative part, we gathered the details of daily PICU hybrid rounds in terms of times, number of HCWs, and type of files shared through Zoom. For the qualitative part, open-ended questions were used.

**Main Results:** The physical round took statistically significantly less time (34.68 ± 14.842 min) as compared with the Zoom round (72.45 ± 22.59 min), *p* < 0.001. The most shared component in the virtual round was chest X-rays (93.5%). Thirty-one HCWs participated in focus group discussions and were included in the analysis. Some of the HCWs' perceived advantages of the hybrid rounds were enabling multidisciplinary discussions, fewer round interruptions, and practicality of virtual discussions. The perceived challenges were the difficulty of the bedside nurse attending the virtual round, decreased teaching opportunities for the trainees, and decreased interactions among the team members, especially if video streaming was not utilized.

**Conclusions:** Multidisciplinary hybrid virtual and physical clinical rounds in the PICU were perceived as feasible by HCWs. The virtual rounds decreased the physical contact between the HCWs, which could decrease the possibility of SARS-CoV-2 spread among the treating team. Still, several components of the hybrid round should be optimized to facilitate the virtual team-members' interactions and enhance the teaching experience.

## Introduction

SARS-CoV-2 infections continue to surge with more than 140 million confirmed cases of COVID-19, including more than 2 million deaths, as reported to the World Health Organization (1). With the second and third waves surging in multiple countries and several SARS-CoV-2 variants posing more challenges, the healthcare system needs more innovations to mitigate the surge in cases and protect the healthcare workers (HCWs) on the front lines (2, 3). Some healthcare systems emphasize the importance of infection prevention for the HCWs even outside the clinical areas due to their vital value (4).

Social distancing is one of the pillars of infection control measures, which may seem difficult to apply in daily hospital rounds, during which the whole healthcare team meets at the bedside, discussing the new clinical developments and best management approach for each patient. Previous research shows that most activities on attending rounds do not actually need to take place at the bedside (5). Another similarly important aspect of these bedside rounds is the clinical teaching and multidisciplinary interactions that are vital to the ongoing process of perfecting the healthcare professionals of the future with better utilization of healthcare resources, which also does not necessarily require close gathering at the bedside (6).

After successfully implementing the virtual handover process of our pediatric intensive care unit (PICU) patients during the COVID-19 crisis (7), we planned to explore the feasibility of a hybrid morning daily clinical round in the PICU that was implemented in September 2020 (Appendix 1). This pilot study explores whether this hybrid round style decreased the timing of the physical proximity between the HCWs. Another aim was to facilitate multidisciplinary team discussions, especially when several team members were not attending the hospital daily during the pandemic crisis.

## Method

### Study Design

This study is a mixed quantitative and qualitative deductive thematic content analysis of the narrative responses from various HCWs in the PICU. The main aim of the qualitative component is to seek future potentials for this novel, hybrid-style PICU round application. We choose the focus group (F.G.) method to clarify issues that may be difficult to raise in one-to-one interviews, such as dissatisfaction with services provided (8).

### Setting

The HCWs of the PICU at King Saud University Medical City (KSUMC) include six consultants, eight registrars, four to six training residents, two PICU fellows, 45 nurses, one pharmacist, one clinical dietician, one social worker, and rotating respiratory therapists. The PICU team serves 15 ventilated beds.

The hybrid rounds were newly implemented on May 15, 2020. Their structure consisted of starting the daily clinical round with a Zoom® meeting involving all the members of the PICU, including the physicians, bedside nurses, pharmacist, dietician, respiratory therapist, and social worker. The Zoom® meeting was mainly devoted to discussing all the patients, including all the clinical data from all involved disciplines; the suggested management plan; and the educational aspect needed for specific clinical issues. After the virtual meeting, the on-call team, bedside nurses, and whoever is needed in the PICU attends the physical rounds at the bedside for the issues requiring addressing there and counseling the parents about their child's status.

### Sampling and Recruitment

HCWs from various PICU backgrounds were invited to participate in this F.G. on November 12, 2020.

#### Data Collection

Open-ended questions were used as per Appendix 2.

#### Data Analysis

The first step in the analysis involved reading and familiarization with the participants' range of responses. Categories were established, and two authors (NA and MT) developed codes independently. NA, an expert in qualitative methodology working in family and community medicine, introduced an etic perspective of the topic, and MT, a PICU consultant, introduced an emic perspective.

The developed codes were similar and were discussed before a consensus on the coding frame was established. All themes were *a priori* themes; however, the range of responses under each subtheme was derived from the data. Qualitative data management was conducted using NVivo 10®.

After obtaining institutional review board approval, we invited PICU physicians of KSUMC, who had been working in a hybrid manner using Zoom®, to describe their experience through a qualitative F.G. virtual meeting. Content analysis was used to analyze the participants' responses. The results were used as a part of the quality improvement project and shared with the pediatric department quality committee.

### Statistical Analysis

Descriptive statistics were used to summarize the data. For the categorical data, we used frequencies and descriptive procedures (minimum, maximum, mean, and S.D). A line chart connecting a series of data points with a continuous line is used to show the trend over time.

## Results

### Quantitative Part

**Figure 1:**
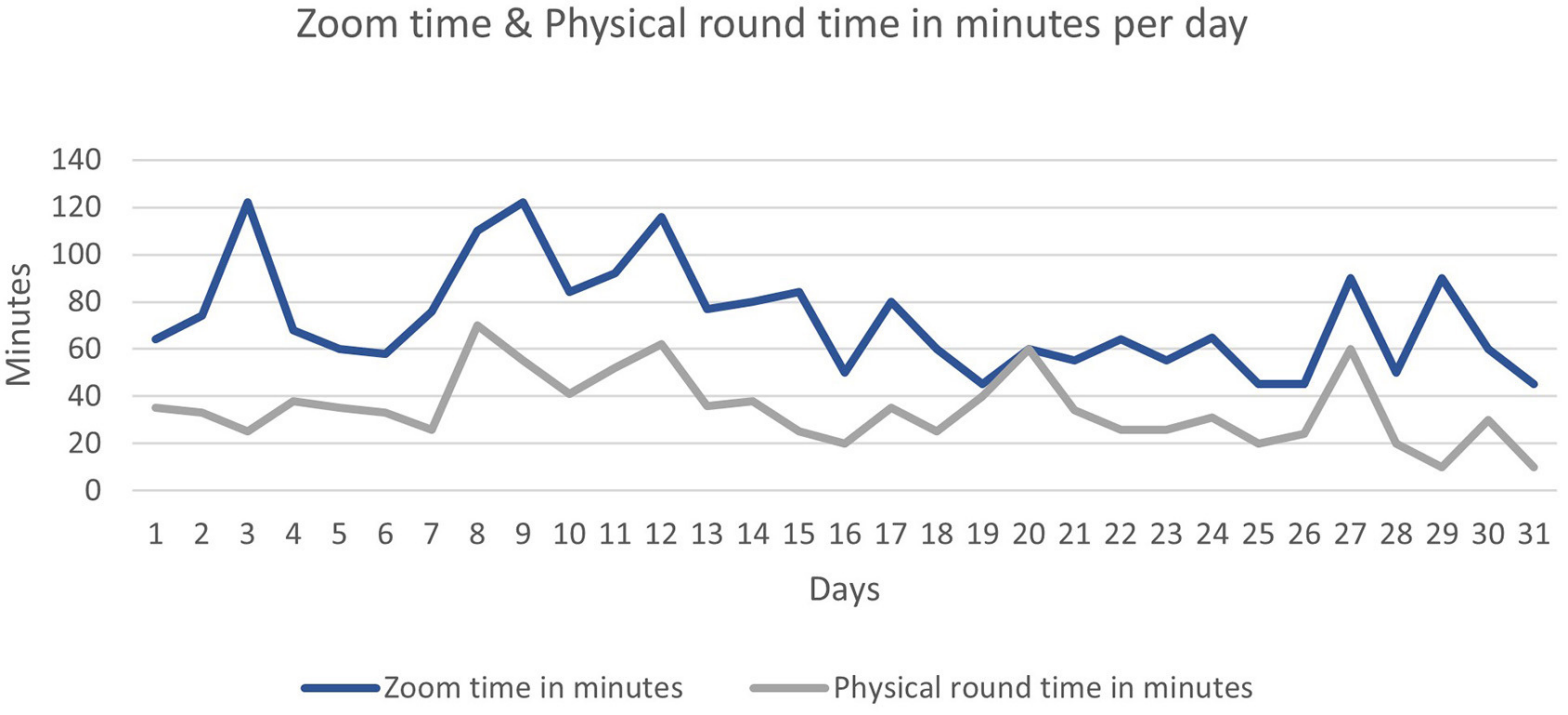
Zoom time and physical round time in minutes per day. *ECG, electrocardiogram; EHR Electronic Health Records; CXR, chest X-rays.

Our analysis shows a clear difference between the time spent during the Zoom® rounds and physical rounds as shown in Table 1. Additionally, over the 1-month pilot study period, the time spent during zoom rounds has dropped from around 60 min in the beginning to around 40 min at the end of the month although the number of patients was almost consistent throughout the month. This trend was also observed for the physical rounds as time spent dropped from 38 to 18 min (Figure 1).

**Table 1:** PICU Zoom round and physical round times (in minutes).

	***N***	**Minimum**	**Maximum**	**Mean**	**Median**	**Std. Deviation**	**Sum**
Zoom time	31	45	122	72.45	65	22.590	2,246
Physical round time	31	10	70	34.68	33	14.842	1,075

The paired-samples *t*-test was used to compare the duration of the round (minutes) between physical and virtual rounds; the analysis shows that the physical rounds required significantly less time (M = 34.68, SD = 14.84) than the virtual Zoom part (M = 72.45, SD = 22.6), *p* < 0.001.

**Figure 2:**
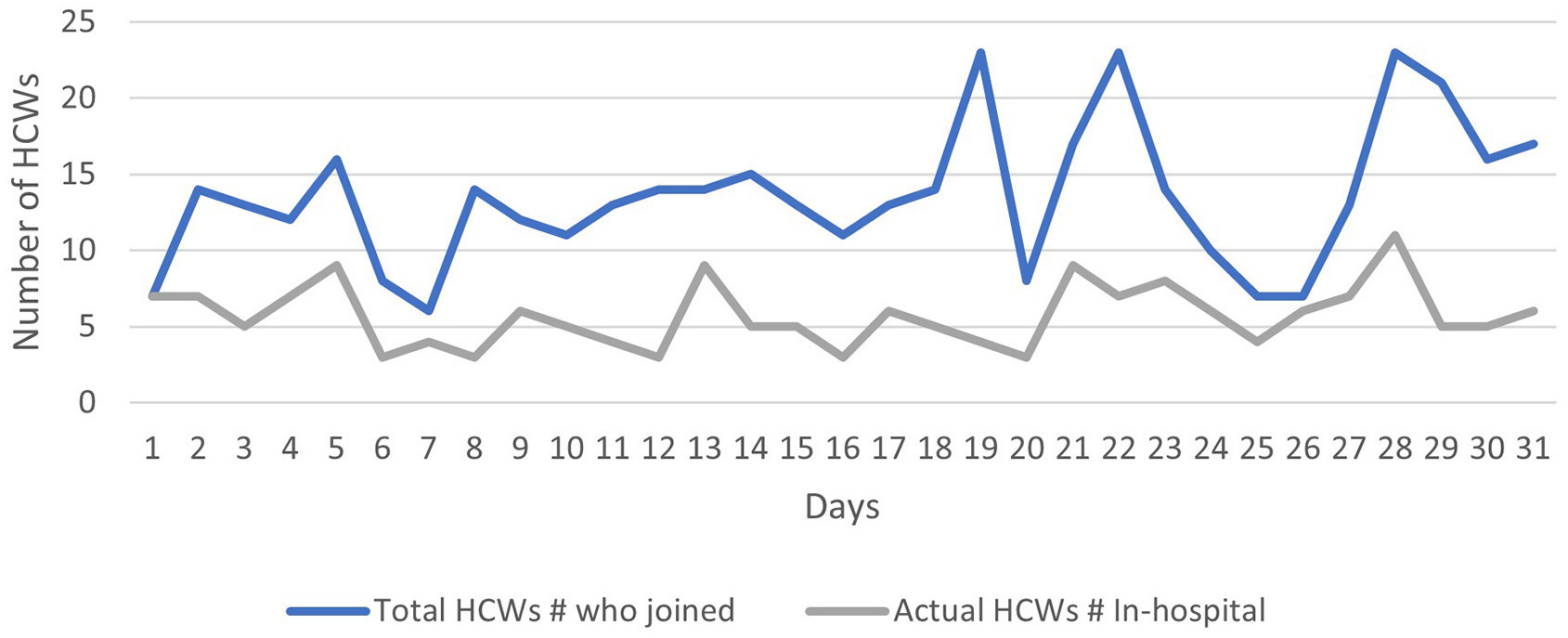
Total number HCWs who joined both the Zoom and the physical (in-hospital) part of hybrid rounds per day.

**Figure 3:**
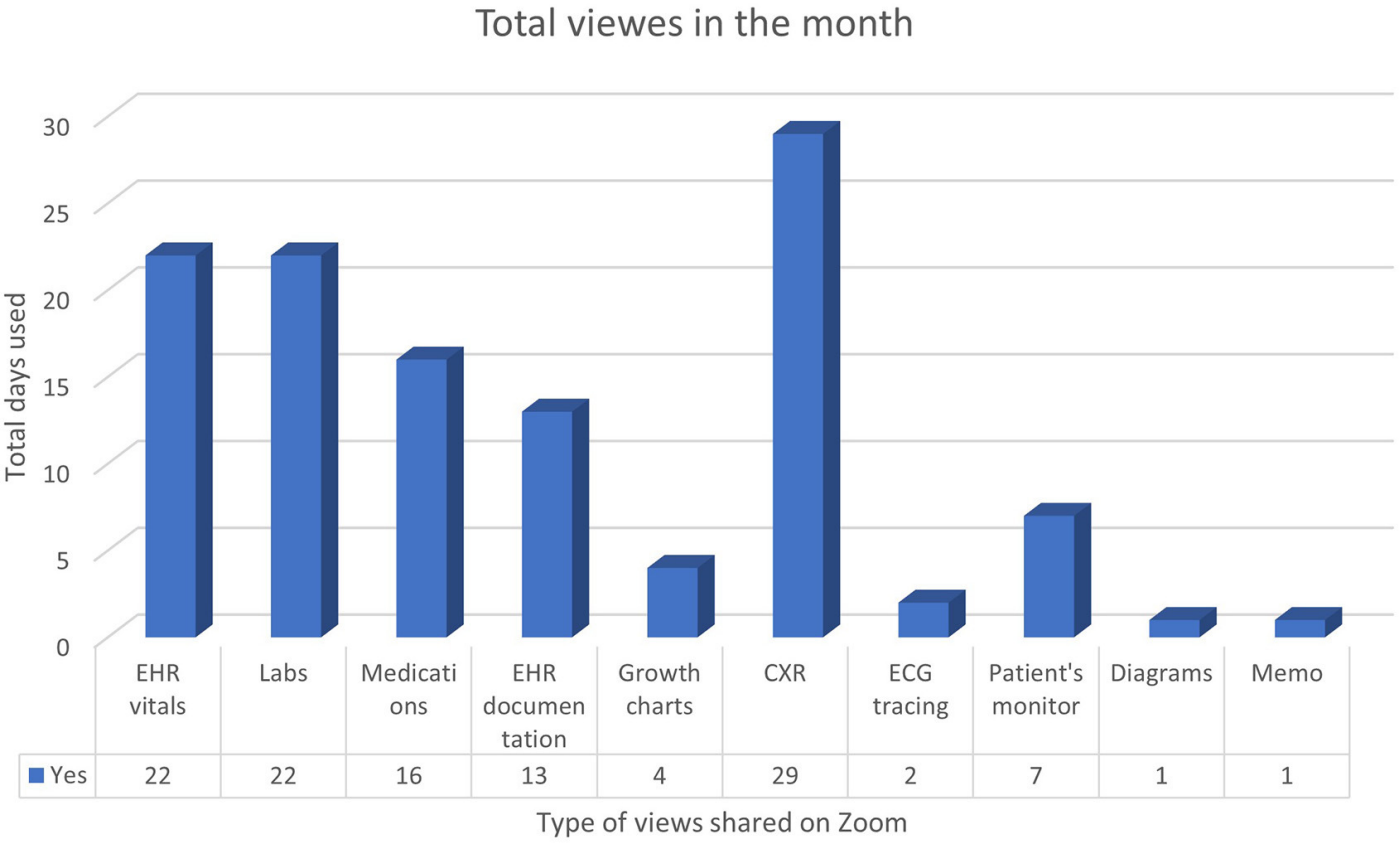
Total views per type shared on the Zoom round over the month. EHR, electronic health records; CXR, chest x-rays.

Regarding the number of staff who attended the hybrid rounds, our results show that the number of HCWs attending the Zoom® part increased steadily over the study period, from seven in the beginning to more than 15 at the end of the month, and the number of the staff who attended the physical (in-hospital) part remained somewhat stable over the study period (five to seven) as shown in Figure 2 and Table 2. During the Zoom meetings, the most commonly shared files were chest X-rays that were shared almost daily (Figure 3).

**Table 2:** Total number of HCWs who joined the Zoom part of the hybrid PICU round and the actual HCWs who joined the subsequent physical round.

	**Minimum**	**Maximum**	**Mean**	**Median**	**Std. Deviation**
Total number of HCWs who joined Zoom part of hybrid rounds	6	23	14	15	4.224
Actual in-hospital HCWs attending the physical part of hybrid rounds	3	21	6	5	2.053

### Qualitative Part

Twelve PICU HCWs joined the F.G.: three consultants, three specialists, two training residents, two nurses, a pharmacist, and a dietician. During the meeting, participants discussed factors that affected their practice as a result of online rounds. The following presents the themes that were discussed during the F.G.

## Perceived Advantages of Hybrid Round

Besides lowering the chances of being infected with COVID-19, participants mentioned other advantages they perceived as a result of using hybrid rounds. The following represents subthemes of perceived advantages.

### Multidisciplinary Meeting

All participants believed that one of the most significant advantages of Zoom meetings is the opportunity to assemble people from different specialties at the same time to discuss patients' conditions.

“The only thing that I think Zoom probably has an advantage in is for the multidisciplinary meetings regarding patients with different subspecialties joining. Otherwise, we do have difficulty arranging a meeting that suits everybody” P10.

The participants appreciated the convenience of inviting colleagues from other disciplines to discuss PICU cases. “You can invite any subspecialty, who could attend with us…if we need to discuss a specific patient for a specific concern” P1.

### Family Involvement

All participants appreciated the benefits they encountered regarding online communication between the PICU team and families. According to them, not having several family members at the bedside made them better focus on their clinical rounds, finish on time, and give families their undivided attention when discussing their children's medical status.

One participant remarked, “Now we focus more and avoid distractions from overcrowding areas…just avoid noisy areas with families gathering or interrupting the round” P8.

Another one added, “We have a special link for the families. Also, that is really very helpful for us because otherwise the families are coming during COVID crisis and interrupting the team dynamics, it is helpful for the PICU workflow” P2.

### The Practicality of Online Meetings

Participants mentioned many points related to the practicality of online rounds. All participants agreed that having online meetings from their offices was more convenient. “It's very convenient that you could be sitting all the time discussing things you could have your cup of tea or coffee in your office while you're in the round” P10.

Four of them also expressed being more efficient after the introduction of online rounds. For example, “My computer is in front of me, so I'm checking the patient during the Zoom round. I can check the labs, check the literature while they discuss the patient's condition…When we finished the rounds, I can promptly put the orders in the EHR. Previously, with the physical rounds, I had to wait until we finish, and then I would go to my office and start doing the orders for the patients, which is more time-consuming” P5.

Another participant added, “For us, Zoom meetings are effective: we can finish our task during the rounds. We can finish the orders swiftly” P11.

## Perceived Challenges of Hybrid Round

Participants in the F.G. addressed some challenges that they faced during hybrid rounds. The following presents the subthemes that emerged from the discussion.

### Nursing Duties

During the F.G., both nurses agreed on the difficulty of keeping up with online rounds and patient care at the same time. However, this is similar to the previous PICU rounds, when the nurses used to attend frequently to the patient's needs while the charge nurse would continue with the round. This was explained by one of the participants: “The charge nurse should be present throughout the hybrid round…And the assigned nurse for each patient before the pandemic will be involved in the rounds when her assigned patient is being discussed. Now, if we are asking nurses to attend the whole Zoom rounds, that could be less time attending their patients?” P5.

A head nurse added, “Sometimes while we attend the Zoom round, something may happen to the patient, and the bedside nurse has to go inside the room immediately…so, when not discussing their cases, there is no point in attending the whole Zoom round” P3.

The time nurses spent on online rounds was sometimes lengthy yet justified from their perspective: “It's different from day to day, but it's around one or one and a half hours. According to the number of patients we have and the severity of the cases and if they may need a lot of discussions or have multiple teaching points” P3.

This challenge experienced by the nurses made some other participants look for a solution to overcome it. A resident commented, “I suggest that we can have sequence numbering of patients that we are discussing. Number one would be the first patient to discuss, can be the sickest one. Number two, the less sick…and like that. The nurse would be able to know the order of when they are discussing her patient and be prepared to log in on time” P6.

Another consultant noted, “So, if they can share with the nurses through one dedicated device with the charge nurse handing it to each assignment nurse. So when we are discussing patient X, the device will be with the nurse assigned to patient X. They would attend for 15–20 min, then they will be focusing on patient care” P12.

### Teaching Opportunities

Both residents within the F.G. were concerned about learning and commented on the effect of hybrid rounds on teaching opportunities. “Previously, there was more discussion and more teaching points to be addressed. I lost that sense in the hybrid rounds” P11.

One participant compared the discussions held at the bedside with those online; she explained, “Usually, bedside teachings were better. There's more discussion and more brainstorming and motivates me…” P8.

She continued, “When things occurred during the round, the whole team is involved, to reflect on what happened…and how we manage it…This aspect of the teaching: we lost because we are sitting away from the patients during the round, just to be able to focus more and avoid all distractions” P8.

Furthermore, residents commented on the effect of hybrid rounds on “X-ray rounds,” one participant explained, “Previously, we used to start our PICU rounds with X-ray rounds, for 20 min discussing only all radiological exams of PICU patients. Nowadays, during COVID, we're having Zoom rounds, we will show some X-rays, we will share the screen for the X-ray and sometimes pass it quickly” P5.

Participating consultants approved their residents' concerns and explained their point of view on teaching using online methods. One of the consultants noted, “Sometimes, I don't feel motivated enough to teach during the rounds compared to the usual rounds. You can easily change your tone. You see the facial expressions you see who want to get more of your teaching. So that would all motivate you to give more” P10.

Another consultant added, “It [bedside rounds] used to be much more interactive with the application of knowledge rather than just didactic lecturing online or just answer the questions rather than building confidence into the solid background of the theoretical and practical approaches…people get “pulled away” from teaching if they are just facing a screen instead of reflecting on a patient look or patient monitor or ventilator or even something in the equipment has changed our approach to the medications. It's [face-to-face teaching] totally much more engaging” P9.

Furthermore, teaching was affected by the number of residents attending during the pandemic: “During COVID, it's really affected bedside teaching, especially for the residents. Daily, the hospital decreased the number of residents attending daily to enhance social distancing. Only three residents (from six or seven) attending inside the PICU. So, the clinical teaching is really compromised during this time” P12.

Another participant added, “Still, some residents outside the hospital had the chance to participate in the Zoom round, so that could be a chance for more educational interactions even when they were not there in the physical rounds” P9.

### Discussions and Interactions

Although it was recommended that participants use their cameras during virtual rounds, they mostly did not.

According to a consultant, “We tell people inside the hospital: please turn on your camera to make more interactions, especially for teaching or discussions. But mostly, they are not listening; they just open their mics when they decide to talk” P4.

During the rounds, proper engagement and interactions from the team members were limited for reasons related to not seeing the speakers. A participant noted, “The engagement and reading people body languages, getting people attention, focusing on what people need, reflecting on and building on ideas gradually as a group. This is usually done much better face-to-face, compared to just online…The online style maybe limiting the team's interactions” P9.

A consultant noted, “I think this sometimes may compromise a patient's care if the nurse doesn't speak up during the online meeting. During most of the online meetings, the nurses' interaction seemed less compared as to the face-to-face or physical rounds” P9.

Another participant added, “The Zoom becomes sometimes boring. Sorry to say that because, as with lectures, hearing is not like hearing and seeing, so you cannot fully concentrate and interact like when you are in physical rounds. You will also feel distracted since you are in your office; you can do other things while you are attending the round” P7.

### The Need to See Patients in the PICU by All Team Members

There were different opinions among the care team members about the utility of the virtual rounds as relates to the patient care. Physicians within the group thought that online meetings could not be blamed for compromising patient care as explained in the following quotes:

“Physicians who are assigned with the patients, they are coming to the bedside” P3.

“There are several physicians who are already available in the unit for the patients, so their care is not affected at all” P2.

On the other hand, the nutritionist thought that she needs to see the patient to provide a better service. She illustrated, “I go look at the patient and see if he/she is wasted or overweight and well-nourished. Now I miss those things because I don't go to the PICU as before COVID. So, I depend on the numbers, like height and weight, which are sometimes not accurate, so I must check with the doctors and nurses about the patient's physical appearance” P5.

### Role of R.T.s in the Online Rounds

Despite participants' acknowledgment of the convenience of multidisciplinary meetings using online methods, this did not facilitate involving R.T.s, whose roles were perceived as crucial for patient care quality in the PICU. One of the consultants clarified, “Before we are having dedicated R.T. to the PICU. Yeah. Now, during the COVID crisis, the R.T.s are shared with two or three other units, there is more demand for their services during the pandemic. It's mainly a respiratory pandemic. So that's a problem…it was another challenge to invite them because the R.T. has very valid points to raise when we talk about mechanical ventilation. We need them for the full respiratory management of these children” P9.

## Discussion

In this pilot study, the physical PICU clinical round time was significantly less than the Zoom component. Close contact with SARS-COV-2 carriers becomes riskier as more time is spent in such encounters. The Center for Disease Control and Prevention (CDC) reported in its Morbidity and Mortality Weekly Report (MMWR) on April 14, 2020, about HCWs who developed COVID-19 after having longer durations of exposure to the index COVID-19 patient (9). These findings underscore the heightened COVID-19 transmission risk associated with prolonged, unprotected patient contact. The CDC recently reported that recurring brief encounters could lead to COVID-19 transmission (9). This could be an additional risk for HCWs in the acute care areas who manage COVID-19 suspected patients. Furthermore, asymptomatic SARS-COV-2 infection was reported in 14.3% of HCWs, implying that strict infection measures among HCWs are needed to reduce transmission risks to other HCWs and patients (10).

The implementation of the Zoom hybrid round was feasible yet challenging in our PICU setting. The utilization of technology as a novel communication tool during the pandemic and tele-ICU was advocated in recent literature (7, 11, 12). During times of physical distancing, HCWs may find it helpful to use videoconferencing services to sustain professional meetings and continue educational activities using online platforms (11). Recent policy changes in telemedicine during the COVID-19 pandemic have generated technology-based clinical tool opportunities, which could help conserve personal protective equipment (PPE) and protect HCWs (13). Such a new approach was labeled electronic PPE (ePPE), which would maintain clinical services, preserve the actual PPE, and keep HCWs safe (13).

Virtual rounds in our pilot period replaced 60% of the physical round. The estimated time saved was utilized to enhance and augment the discussion about the patients' conditions, laboratory findings, and teaching. Such rounds are essential aspects of the education and teaching of medical students, interns, and residents, allowing them to understand key information and develop clinical reasoning (14). Virtual rounds may also decrease the embarrassment that students may have due to the presence of patients. However, one drawback is the lack of patient–learner interactions (14, 15). Other studies show that virtual rounds specifically designed to manage COVID-19 patients had a favorable assessment by patients and learners (16). It is also shown that virtual teaching is effective and may further enhance education by the availability of different specialties (17).

Our hybrid clinical round setting allowed multidisciplinary teams, reaching up to 23 HCW at the same time while maintaining social distancing. In a perspective piece on remote pediatric healthcare delivery during the pandemic, researchers highlighted the different variations and innovations in adaptation and called for integrating telemedicine and virtual health (18). Our hybrid approach can be adapted and validated by other PICUs in which the number of HCWs may exceed ours and when physical distancing may not be feasible. Although vaccination of HCWs is being rolled out with excellent efficacy (19, 20), the emergence of mutant variants (21–23) will continue to challenge HCWs and still imply continued universal masking and physical distancing (24).

Our study participants commented about the need for flexibility in the hybrid round to allow for patient care as needed. ICU nursing staff are under unprecedented pressure during the pandemic and show resilience and continued patient care despite all stresses and pressure (25); this type of hybrid round would help alleviate some of that pressure without compromising patient care.

Some overwhelmed clinical services, such as R.T.s, were unable to join the Zoom part of the rounds in our setting. With the overwhelming number of critical care patients requiring respiratory support, a surge in demand for respiratory therapists was seen with safety, treatment, and staffing recommendations published (26) and evaluating tools on R.T. extended comfort with mechanical ventilation during the pandemic, a hybrid type of round may help alleviate such pressure on R.T.s allowing them to have more time to tend to their patients (27).

This study shows that the most shared items during the virtual clinical round were radiography, EHR vitals, and laboratory data. The ability to share radiographs is an essential aspect of the Zoom clinical rounds. In one study, radiology residents could transition the teaching conference and educational lectures entirely to a virtual format (28). Of course, as indicated previously, one possible extension of this feature is that trainees may seek input from more senior physicians using Zoom methods (28). This feature was highly appreciated by the residents who were involved in our study. In addition, it is very convenient to read out or share screens to discuss other patients' related data, such as laboratory data and vital signs either as data points or utilizing any graphic presentations, provided the patient's confidentiality is maintained with Zoom's end-to-end encryption (29).

Although telehealth has multiple advantages, it also has its pitfalls. One such pitfall is that physical examination might not be optimal, especially for new physicians. In one study, telemedicine demonstrated poor agreement with an in-person examination of patients with tonsilitis (30). Few studies are addressing the validity of virtual examinations (31). Because it is likely that telemedicine for examination will continue beyond the pandemic, some studies are looking at proposals for such examinations (32). This requires additional skills of the individuals using telemedicine, including robust communication skills and the ability to perform such examinations remotely and accurately.

Participants suggest that clinical teaching for the training residents was compromised during COVID-19, especially for those outside the hospital. The Zoom round seemed to have both negative and positive impacts on the teachings but needs to be optimized. It is crucial to build telemedicine into the residency program's curriculum and to expose medical students to the advantages and disadvantages of telemedicine (33). In addition, it is essential to address technical proficiency, history taking, examination skills, and communication (34). In addition, it is important to combine both telemedicine and in-person clinical rounds as a hybrid activity. A pilot study of the use of telemedicine in primary care shows general acceptability with logistics and other concerns. It is important to note the exposure to telemedicine by medical students, and 17.4% of students had prior telemedicine exposure (35). Such integration of telemedicine into medical education is of paramount importance (36).

The presence of the family during rounds and sharing decisions will increase family satisfaction during admission and may enhance the communication between the treating team and parents (37). However, frequent parental interruptions of the PICU round increases the round time and affects the team dynamics (37, 38). Hybrid rounds started initially before in-person rounds, and this can gather information about the patient and sharing decisions among medical team, providing a teaching opportunity for the rotators without interruption from the family (39). Still, the possibility of a decrease in the quality of face-to-face interactions between the residents could affect the interactive family scenarios in the PICU (40).

## Limitations

This single-center experience needs to be validated in other settings. Training may be more challenging in other hospitals as our PICU team was already using videoconferencing by Zoom for patient handover. Although we did notice a decrease in the time of the hybrid round over the 1-month study period for the same number of PICU patients, this observation might still point to the team's learning curve, which has improved over time with the new hybrid system of daily rounds. However, this study was not designed to examine the HCWs' learning curve, and reporting learning curves in health profession research is deficient and often underutilizes their desired properties (41). Also, in our F.G., representativeness was not a goal for the qualitative part of this study. Therefore, further research could benefit from getting more nurses' input on the nature of their involvement with their PICU patients and families.

In addition, further research is required to focus on predefined patient outcomes in a multicenter prospective trial. The current pilot study was intended to check the feasibility of this hybrid style, and the PICU rounds' time depends on multiple factors, such as the number and complexity of the PICU patients and the interdisciplinary team dynamics, and other factors that may be different from day to day. Thus, it could be best to examine the direct effect of a virtual clinical round on reducing the duration of the physical rounds in future research.

## Conclusion

Hybrid-style rounds in the PICU were feasible and decreased the timing of the physical round. The virtual component of the daily hospital rounds facilitated multidisciplinary discussions and trainees' interactions both inside and out of the hospital setting. However, there were concerns about the quality of teaching and team members' interactions, especially when the cameras were not used. More studies are needed to explore if the virtual part of the clinical round is best suited for the patients' management and HCWs' experience.

## Data Availability Statement

The original contributions presented in the study are included in the article/Supplementary Materials, further inquiries can be directed to the corresponding author/s.

## Ethics Statement

The studies involving human participants were reviewed and approved by IRB, College of Medicine, King Saud University, Riyadh, Saudi Arabia. Written informed consent for participation was not required for this study in accordance with the national legislation and the institutional requirements.

## Author Contributions

M-HT, AAlhb, NA, JA-T, FAlj, AA-E, MB, and AAs conceptualized the study, analyzed the data, and wrote the manuscript. FAls, AJ, MA, OE, and RT contributed to the study design, collected, analyzed, interpreted data, and edited the manuscript. KA contributed to the study design, interpretation, and edited the manuscript. AB, AAlhe, and RH interpreted the data and finalized the manuscript. All authors contributed to drafting, reviewing, and approving the final version of the manuscript.

## Conflict of Interest

The authors declare that the research was conducted in the absence of any commercial or financial relationships that could be construed as a potential conflict of interest.

## Publisher's Note

All claims expressed in this article are solely those of the authors and do not necessarily represent those of their affiliated organizations, or those of the publisher, the editors and the reviewers. Any product that may be evaluated in this article, or claim that may be made by its manufacturer, is not guaranteed or endorsed by the publisher.

## Supplementary Material

Appendix A1 Details of the PICU hybrid-style round.

Appendix A2 The F.G. themes and questions.

## Abbreviations

CDC: Centers for Disease Control and Prevention
COVID-19: coronavirus disease 2019
EHR: electronic health records
ePPE: electronic personal protective equipment
KSUMC: King Saud University Medical City
PICU: Pediatric Intensive Care Unit
RT: respiratory therapist
WHO: World Health Organization.

## References

[1] Organization WH. WHO Coronavirus (COVID-19) Dashboard. (2021). Available online at: https://covid19.who.int/ (accessed April 18, 2021).

[2] Lancet T. COVID-19: protecting health-care workers. Lancet. (2020) 395:922. 10.1016/S0140-6736(20)30644-9 32199474 PMC7138074

[3] Nguyen LH Drew DA Graham MS Joshi AD Guo CG Ma W. Risk of COVID-19 among front-line health-care workers and the general community: a prospective cohort study. Lancet Public Health. (2020) 5:e475–83. 10.1101/2020.04.29.20084111 32745512 PMC7491202

[4] Ling L Wong WT Wan WTP Choi G Joynt MG. Infection control in non-clinical areas during the COVID-19 pandemic. Anaesthesia. (2020) 75:962–3. 10.1111/anae.15075 32267964 PMC7262124

[5] Stickrath C Noble M Prochazka A Anderson M Griffiths M Manheim J. Attending rounds in the current Era: what is and is not happening. JAMA Internal Med. (2013) 173:1084–9. 10.1001/jamainternmed.2013.6041 23649040

[6] Dutton RP Cooper C Jones A Leone S Kramer ME Scalea MT. Daily multidisciplinary rounds shorten length of stay for trauma patients. J Trauma Acute Care Surg. (2003) 55:913–9. 10.1097/01.TA.0000093395.34097.56 14608165

[7] Temsah M-H Abouammoh N Ashry A Al-Eyadhy A Alhaboob A Alsohime F. Virtual handover of patients in the pediatric intensive care unit during the Covid-19 crisis. J Multidiscipl Healthcare. (2021) 14:1571–81. 10.2147/JMDH.S310028 34211276 PMC8241813

[8] Green J. Qualitative methods. Community Eye Health. (1999) 12:46–7. 17492000 PMC1706011

[9] CDC. COVID-19 in a Correctional Facility Employee Following Multiple Brief Exposures to Persons with COVID-19 – Vermont. (2020). Available online at: https://www.cdc.gov/mmwr/volumes/69/wr/mm6943e1.htm (accessed April 18, 2021). 10.15585/mmwr.mm6943e1 PMC7640999 33119564

[10] Abdelmoniem R Fouad R Shawky S Amer K Elnagdy T Hassan WA. SARS-CoV-2 infection among asymptomatic healthcare workers of the emergency department in a tertiary care facility. Journal of Clinical Virology. (2021) 134:104710. 10.1016/j.jcv.2020.104710 33276180 PMC7694465

[11] Garfin DR. Technology as a coping tool during the coronavirus disease 2019 (COVID-19) pandemic: Implications and recommendations. Stress Health. (2020) 36:555–9. 10.1002/smi.2975 32762116 PMC7436915

[12] Chandra S Hertz C Khurana H Doerfler EM. Collaboration between tele-ICU programs has the potential to rapidly increase the availability of critical care physicians-our experience was during coronavirus disease 2019 nomenclature. Crit Care Explorat. (2021) 3:e0363. 10.1097/CCE.0000000000000363 33786439 PMC7994031

[13] Turer RW Jones I Rosenbloom ST Slovis C Ward JM. Electronic personal protective equipment: A strategy to protect emergency department providers in the age of COVID-19. J Am Med Inform Assoc. (2020) 27:967–71. 10.1093/jamia/ocaa048 32240303 PMC7184500

[14] Hagana A Behranwala R Aojula N Houbby N. Digitalising medical education: virtual ward rounds during COVID-19 and beyond. BMJ. (2020) 7:742. 10.1136/bmjstel-2020-000742 PMC8936893 35516826

[15] Vogel D Harendza S. Basic practical skills teaching and learning in undergraduate medical education - a review on methodological evidence. GMS J Med Educ. (2016) 33:Doc64. 10.3205/zma001063 27579364 PMC5003143

[16] Hofmann H Harding C Youm J Wiechmann W. Virtual bedside teaching rounds with patients with COVID-19. Med Educ. (2020) 54:959–60. 10.1111/medu.14223 32403185 PMC7273015

[17] Wilcha RJ. Effectiveness of virtual medical teaching during the COVID-19 crisis: systematic review. JMIR Med Educ. (2020) 6:e20963. 10.2196/20963 33106227 PMC7682786

[18] Badawy SM Radovic A. Digital approaches to remote pediatric health care delivery during the COVID-19 pandemic: existing evidence and a call for further research. JMIR Pediatr Parent. (2020) 3:e20049. 10.2196/20049 32540841 PMC7318926

[19] Hall VJ Foulkes S Saei A Andrews N Oguti B Charlett A. COVID-19 vaccine coverage in health-care workers in England and effectiveness of BNT162b2 mRNA vaccine against infection (SIREN): a prospective, multicentre, cohort study. Lancet. (2021) 397:1725–35. 10.1016/S0140-6736(21)00790-X 33901423 PMC8064668

[20] Thompson MG Burgess JL Naleway AL Tyner HL Yoon SK Meece J. Interim estimates of vaccine effectiveness of BNT162b2 and mRNA-1273 COVID-19 vaccines in preventing SARS-CoV-2 infection among health care personnel, first responders, and other essential and frontline workers - eight locations US. December 2020-March 2021. MMWR Morb Mortal Wkly Rep. (2021) 70:495–500. 10.15585/mmwr.mm7013e3 33793460 PMC8022879

[21] Hacisuleyman E Hale C Saito Y Blachere NE Bergh M Conlon EG. Vaccine Breakthrough Infections with SARS-CoV-2 Variants. N Engl J Med. (2021) 384:2212–8. 10.1056/NEJMoa2105000 33882219 PMC8117968

[22] Cavanaugh AM Fortier S Lewis P Arora V Johnson M George K. COVID-19 outbreak associated with a SARS-CoV-2 R.1 lineage variant in a skilled nursing facility after vaccination program - Kentucky, March 2021. MMWR Morb Mortal Wkly Rep. (2021) 70:639–43. 10.15585/mmwr.mm7017e2 33914720 PMC8084128

[23] Teran RA Walblay KA Shane EL Xydis S Gretsch S Gagner A. Postvaccination SARS-CoV-2 infections among skilled nursing facility residents and staff members - Chicago, Illinois, December 2020-March (2021). MMWR Morb Mortal Wkly Rep. (2021) 70:632–8. 10.15585/mmwr.mm7017e1 33914721 PMC8084122

[24] CDC. Interim Public Health Recommendations for Fully Vaccinated People. (2021). Available online at: https://www.cdc.gov/coronavirus/2019-ncov/vaccines/fully-vaccinated-guidance.html#print (accessed May 30, 2021).

[25] Thusini S. Critical care nursing during the COVID-19 pandemic: a story of resilience. Br J Nurs. (2020) 29:1232–6. 10.12968/bjon.2020.29.21.1232 33242276

[26] Vitacca M Carone M Clini EM Paneroni M Lazzeri M Lanza A. Joint statement on the role of respiratory rehabilitation in the COVID-19 crisis: the Italian position paper. Respiration. (2020) 99:493–9. 10.1159/000508399 32428909 PMC7316664

[27] Roberts KJ Johnson B Morgan HM Vrontisis JM Young KM Czerpak E. Evaluation of respiratory therapist extender comfort with mechanical ventilation during COVID-19 pandemic. Respir Care. (2021) 66:199–204. 10.4187/respcare.08459 33323412

[28] Li CH Rajamohan AG Acharya PT Liu CJ Patel V Go JL. Virtual read-out: radiology education for the 21st century during the COVID-19 pandemic. Acad Radiol. (2020) 27:872–81. 10.1016/j.acra.2020.04.028 32386950 PMC7252195

[29] Yuan ES. End-to-End Encryption Update. Zoom Blog. (2020). Available online at: https://blog.zoom.us/end-to-end-encryption-update/ (accessed July 26, 2020).

[30] Akhtar M Van Heukelom PG Ahmed A Tranter RD White E Shekem N. Telemedicine physical examination utilizing a consumer device demonstrates poor concordance with in-person physical examination in emergency department patients with sore throat: a prospective blinded study. Telemed J E Health. (2018) 24:790–6. 10.1089/tmj.2017.0240 29470127 PMC6205037

[31] Ansary AM Martinez JN Scott DJ. The virtual physical exam in the 21st century. J Telemed Telecare. (2019) 2019:1357633x19878330. 10.1177/1357633X19878330 31690169

[32] Benziger CP Huffman MD Sweis RN Stone JN. The telehealth ten: a guide for a patient-assisted virtual physical examination. Am J Med. (2021) 134:48–51. 10.1016/j.amjmed.2020.06.015 32687813 PMC7368154

[33] Jumreornvong O Yang E Race J Appel J. Telemedicine and medical education in the age of COVID-19. Acad Med. (2020) 95:1838–43. 10.1097/ACM.0000000000003711 32889946 PMC7489227

[34] Lawrence K Hanley K Adams J Sartori DJ Greene R Zabar S. Building telemedicine capacity for trainees during the novel coronavirus outbreak: a case study and lessons learned. J Gen Intern Med. (2020) 35:2675–9. 10.1007/s11606-020-05979-9 32642929 PMC7343380

[35] Fleming DA Riley SL Boren S Hoffman KG Edison KE Brooks SC. Incorporating telehealth into primary care resident outpatient training. Telemed J E Health. (2009) 15:277–82. 10.1089/tmj.2008.0113 19382866

[36] Lee MS Nambudiri V. Integrating telemedicine into training: adding value to graduate medical education through electronic consultations. J Grad Med Educ. (2019) 11:251–4. 10.4300/JGME-D-18-00754.1 31210850 PMC6570439

[37] Grzyb MJ Coo H Rühland L Dow K. Views of parents and health-care providers regarding parental presence at bedside rounds in a neonatal intensive care unit. J Perinatol. (2014) 34:143–8. 10.1038/jp.2013.144 24202296

[38] Ingram TC Kamat P Coopersmith CM Vats A. Intensivist perceptions of family-centered rounds and its impact on physician comfort, staff involvement, teaching, and efficiency. J Crit Care. (2014) 29:915–8. 10.1016/j.jcrc.2014.07.015 25123794

[39] Gupta PR Perkins RS Hascall RL Shelak CF Demirel S Buchholz TM. The effect of family presence on rounding duration in the PICU. Hosp Pediatr. (2017) 7:103–7. 10.1542/hpeds.2016-0091 28104730

[40] Gaulton J Ziegler K Chang E. Virtual practices transform the care delivery model in an intensive care unit during the coronavirus pandemic. NEJM Catalyst. (2020) 1–11. 10.1056/CAT.20.0169

[41] Howard NM Cook DA Hatala R Pusic VM. Learning curves in health professions education simulation research: a systematic review. Simul Healthc. (2021) 16:128–35. 10.1097/SIH.0000000000000477 32675731

