# Supplementary figures and images for: Pediatric Intensive Care Hybrid-Style Clinical Round During COVID-19 Pandemic: A Pilot Study
Source: Front Pediatr. 2021 Aug 19;9:720203. doi: 10.3389/fped.2021.720203 (PMC8417365; doi:10.3389/fped.2021.720203)

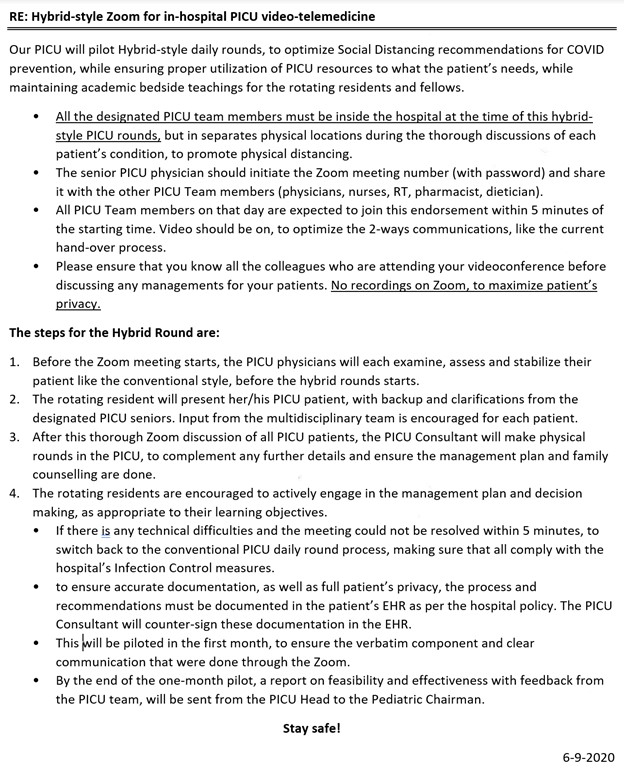

Supplement: Appendix A1 — Details of the PICU hybrid-style round. [file Image_1.JPEG]

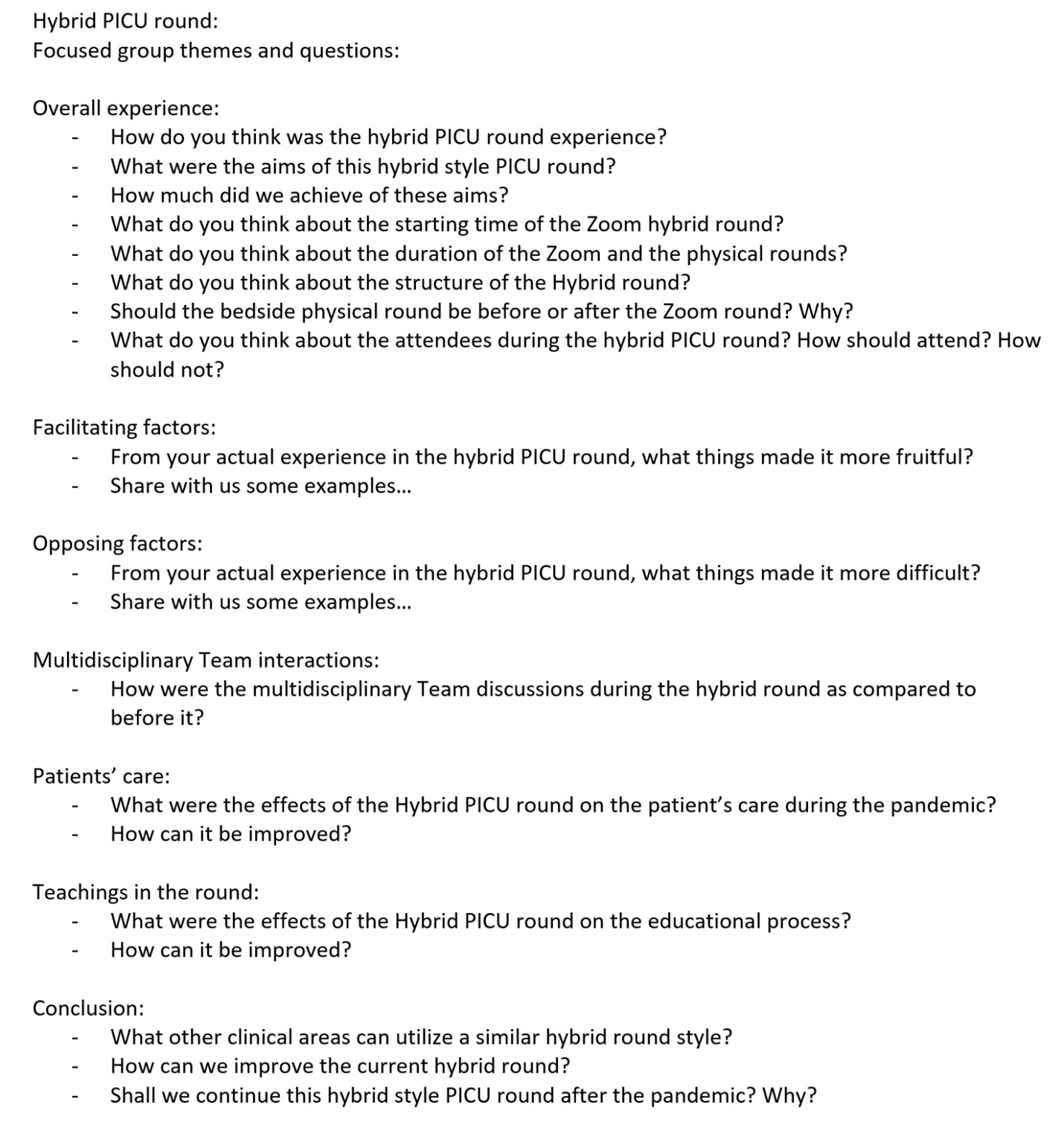

Supplement: Appendix A2 — The F.G. themes and questions. [file Image_2.JPEG]
